# Organizational Health Literacy in Schools: Concept Development for Health-Literate Schools

**DOI:** 10.3390/ijerph19148795

**Published:** 2022-07-20

**Authors:** Sandra Kirchhoff, Kevin Dadaczynski, Jürgen M. Pelikan, Inge Zelinka-Roitner, Christina Dietscher, Uwe H. Bittlingmayer, Orkan Okan

**Affiliations:** 1 Department of Sport and Health Sciences, Technical University of Munich, 80992 Munich, Germany; orkan.okan@tum.de; 2 Department of Health Science, Fulda University of Applied Sciences, 36037 Fulda, Germany; kevin.dadaczynski@pg.hs-fulda.de; 3 Centre for Applied Health Science, Leuphana University of Lueneburg, 21335 Lueneburg, Germany; 4 Austrian National Public Health Institute, 1010 Vienna, Austria; juergen.pelikan@univie.ac.at; 5 Styria Vitalis, 8010 Graz, Austria; inge.zelinka-roitner@styriavitalis.at; 6 Austrian Ministry of Health, 1010 Vienna, Austria; christina.dietscher@gesundheitsministerium.gv.at; 7 Institute of Sociology, University of Education Freiburg, 79117 Freiburg, Germany; uwe.bittlingmayer@ph-freiburg.de

**Keywords:** health literacy, organizational health literacy, health-literate school, health promotion, organizational development

## Abstract

(1) Background: Health literacy is considered a personal asset, important for meeting health-related challenges of the 21st century. Measures for assisting students’ health literacy development and improving health outcomes can be implemented in the school setting. First, this is achieved by providing students with learning opportunities to foster their personal health literacy, thus supporting behavior change. Second, it is achieved by measures at the organizational level promoting social change within the proximal and distal environment and supporting the school in becoming more health-literate. The latter approach is rooted in the concept of organizational health literacy, which comprises a settings-based approach aiming at changing organizational conditions to enhance health literacy of relevant stakeholders. The HeLit-Schools project aims to develop the concept of health-literate schools, describing aspects that need to be addressed for a school to become a health-literate organization. (2) Method: The concept development builds on existing concepts of organizational health literacy and its adaptation to the school setting. (3) Results: The adaptation results in the HeLit-Schools concept describing a health-literate school with eight standards. Each standard depicts an area within the school organization that can be developed for fostering health literacy of school-related persons. (4) Conclusions: The HeLit-Schools concept offers an approach to organizational development for sustainably strengthening health literacy.

## 1. Introduction

Health literacy can be understood as an important competence for dealing with and managing health-related information and knowledge and it is regarded as a core concept of health promotion [1]. It is commonly defined as follows: “Health literacy is linked to literacy and entails people’s knowledge, motivation and competences to access, understand, appraise, and apply health information in order to make judgments and take decisions in everyday life concerning healthcare, disease prevention and health promotion to maintain or improve quality of life during the life course” [2] (p. 3).

The importance of health literacy has never been more apparent than in times of societal developments and challenges such as digitalization [3,4] or health crises such as the current COVID-19 pandemic [5,6]. Although information overload was already a major challenge prior to COVID-19 [7], it was immensely exacerbated by the pandemic, which was accompanied by an information epidemic (“infodemic”). The WHO describes an infodemic as “an over-abundance of information, some accurate and some not, which makes it hard for people to find trustworthy sources and reliable guidance when they need it” [8].

These examples demonstrate the need for personal resources, i.e., health literacy, for managing health-related information. As health literacy is a result of socialization and educational processes [9], international experts argue that it should be marked as a significant educational goal and that measures to strengthen health literacy should be implemented as early as possible in life [10]. Hence, educational settings such as schools can play a critical role in fostering health literacy. Educational interventions at school can teach students basic skills, knowledge, and attitudes at an early age, which, in turn, support them in developing high levels of health literacy during their lives [1]. Furthermore, educational outcomes and health literacy are indirectly linked: while health literacy influences health behaviors and health status, these health outcomes can have a positive impact on educational outcomes. At the same time, education is a determinant of health literacy. In this way, health and education mutually influence each other [4]. Moreover, studies imply that health equity can be increased by health literacy. This is because there is a social gradient for health literacy [11] and because health literacy is considered a critical determinant of health that can be influenced and modified, specifically through different support measures [12,13,14]. The degree of health literacy depends very much on a schoolchild’s social background, which is, as most other factors, determining healthy life years linked to the family of origin’s income and educational background, as indicated by studies on primary [15] and secondary [16] schoolchildren conducted in Germany and internationally [17,18].

In addition to directly addressing students’ personal health literacy, in order to initiate behavior change, and thereby support the development of more healthy behavior, the school organization can be approached about initiating social change by promoting their so-called organizational health literacy, health-literacy responsiveness, or health literacy friendliness [19,20]. This way, strengthening health literacy is not limited to individualistic measures for behavior change but includes measures for systemic social change, including structural and organizational change and development. Such intervention approaches address environmental factors that often have a greater influence on competence, behavior and health [4].

### 1.1. Organizational Health Literacy

Today, health literacy is characterized by its relational nature [21,22]. Whether or not a person possesses a high level of health literacy and can act in a health-literate manner depends on the extent to which their personal abilities and the complexity of the environmental conditions (e.g., the comprehensibility and accessibility of health information or services) fit together. Accordingly, when dealing with health literacy, the respective environment and its requirements must always be considered, making social change an important goal of health literacy, in addition to behavior change. This so-called duality of health literacy is presented in the relational model by Parker and Ratzan (2010) [21]. Recently, Sørensen et al. (2019) [22] added to the model by proposing that it is equally important to consider the competencies within a system (e.g., those of the staff or the facility) in interaction with the complexities of the individuals using the respective system or services.

Hence, strengthening health literacy can be approached in two ways: (i) by educational interventions to directly improve or enhance personal competences (behavior change model), and; (ii) by systemic interventions to modify environmental factors (e.g., accessibility, comprehensibility of health-related information, transparency of services, and organizational capacities) [23], including the responsiveness of organizations [24] (social change model). In the social change model, the organization’s conditions and environment can be altered to be more health-literate and better meet the health literacy needs of individuals. In this way, they function as a facilitator for the management of health-related information, fostering personal health literacy and supporting the development of health-promoting attitudes and behaviors.

The organizational health literacy concept includes both the behavioral and social change approaches, which are addressed within a holistic, settings-based approach to strengthen health literacy. Organizational health literacy originated in the US, where it was developed in order to improve the quality of health services and health promotion in health care and clinical facilities. The concept describes how health-care organizations can be developed and adjusted to be more health-literate. It describes how different organizational changes can be made to better meet patients’ needs (accessibility, transparency, user-friendliness, etc.), to enhance treatment outcomes and communication between professionals and patients, and to positively influence individual health behavior [25]. Thus, an organization can be described as health-literate if it “equitably enable[s] individuals to find, understand, and use information and services to inform health-related decisions and actions for themselves and others.” [26] (p. 1084).

Different approaches to organizational health literacy have been developed in the past [20,27]. The most well-known, and frequently used, concept was presented by the Institute of Medicine (IOM) with an integrated model describing ten attributes of health-literate health-care organizations in 2012 [28]. This concept was further developed in Europe into the Vienna Concept of Health-Literate Hospitals and Healthcare Organizations (V-HLO) [23,29], which was first validated and piloted in Austrian hospitals. It was also translated into French and Mandarin and piloted in Belgian and Taiwanese hospitals [30], later being developed into an International Self-Assessment Tool for Organizational Health Literacy (Responsiveness) of Hospitals (OHL-Hos) by an international working group of the international network of Health Promoting Hospitals and Health Services (HPH).

Similarly to health-care and social-care organizations, for which the organizational health literacy approach is already being used [23,31,32], schools can be understood as organizations. In this sense, the concept of the health-literate organization seems transferable. With regard to schools or educational settings in general, work is being undertaken by use of similar concepts in the US [33], Australia [24,34,35], and Austria [36]. A school is a health-literate organization if its organizational capacities, structures, cultures and processes contribute to developing, practicing, and strengthening personal health literacy for everyone at or related to the school [37]. Previous work in Austria suggests that holistic or systemic approaches and organizational development are more comprehensive and, therefore, more sustainable and cost-effective than individual measures promoting health literacy [23,25,29]. Translated to the school setting, this means that measures should address both behavioral and social change driven by the entire school organization, the school environment, and key stakeholders, but should also include improvements of the core functions of the school, i.e., learning arrangements, opportunities, and motivation, as well as fostering skills of students and staff. In addition, addressing the organizational development of schools can contribute to closing the social gap between schoolchildren from socially disadvantaged and underserved backgrounds and those from privileged backgrounds, as they experience encouragement and educational support from early childhood onwards. A change for health-literate schools must, therefore, include a change in the educational system itself in order to make it more equitable for all.

### 1.2. HeLit-Schools Project

The “Health-Literate Schools” research project (“HeLit-Schools”) was launched in 2019 to explore and use the potential a holistic and systemic approach to organizational development of schools regarding health literacy can have. The project is funded by the German Federal Ministry of Health (2019–2023) and is being carried out in the German school context by researchers from Bielefeld University and the Technical University of Munich.

The main aims of the HeLit-Schools project are to adapt the organizational health- literacy concept for the school setting, to develop a self-assessment questionnaire, and to apply this assessment tool in an online survey to measure schools` organizational health literacy for the first time [37,38,39]. The project is based on a mixed-method study design, combining qualitative research methods (participatory workshops and interviews with central stakeholders, e.g., teachers and principals) and quantitative research methods (online survey to assess organizational health literacy of schools). Organizational health literacy contributes, and is linked to, school-based organizational development, whereby organizational development is described as a process-oriented, holistic approach with a highly systemic character, long-term-oriented planning, and continuous development of the school organization [40]. Its own members are involved in development and changes in the organization and its environmental features and processes. These members are central actors in planning, realization, and monitoring of these developments. Through this approach, the structures, frameworks, networks, and collaborations of the school can be assessed, reflected upon, and changed to be more beneficial for health-literacy promotion [38,39].

Additionally, the project aims at promoting the implementation and evaluation of this concept in the school setting. In order to achieve these goals, (i) practical tools are created for supporting schools in becoming more health-literate; e.g., a guideline for implementation, a toolbox of existing teaching materials to enhance personal health literacy in school, and the aforementioned self-assessment tool for measuring a school’s organizational health literacy. (ii) In terms of evaluation, a measurement of organizational health- literacy of schools based on the HeLit-Schools concept will be conducted to gather empirical data and review the conceptualization. (iii) Considering that the organizational health literacy approach represents a whole-of-school or settings-approach, and that the WHO-driven health promoting school framework [41] is already being implemented in selected German schools, including state networks [42,43], the HeLit-Schools project also aims to interlink both concepts and avert conflict between the two whole-of-school approaches that promote health. (iv) Finally, another important goal was to launch a nationwide network for school health literacy. This was realized in 2021 with the creation of the German Alliance Health Literacy in Schools [44] in collaboration with researchers, practitioners, and policy actors. The Alliance brings professionals, networks, and interested parties together for collaboration on health literacy in schools, serving as a network hub to support the inclusion of health literacy in the school setting and in educational policies.

The objective of this article is to present the development of a concept for organizational health literacy for schools that has taken place within the HeLit-Schools research project. First, the development process that builds on adaptation work will be presented, followed by an introduction of the Health-Literate Schools concept (HeLit-Schools concept) as a result of the adaptation work. On the one hand, this concept provides answers as to what characterizes a health-literate school. On the other hand, it specifies content-related and school-specific organizational aspects that must be addressed in order to develop a school into a health-literate organization, which helps its students, staff, and those in the school environment to improve their health literacy and, in turn, their health.

## 2. Materials and Methods

### 2.1. Concept Development: Adaptation of Existing Concepts

Concept development was informed by earlier work on organizational health literacy in Austria and builds mainly upon two concepts developed for different settings: the original Vienna Concept of Health-Literate Hospitals and Healthcare Organizations (V-HLO) [23,45] and the concept of Health-Literate Extracurricular Youth Work of the Austrian Network for Open Youth Work and the Austrian Youth Information Center (“bOJA/BÖJI”) [31,46]. The V-HLO concept partly builds upon the “10 Attributes of Health Literate Health Care Organizations” [28] developed in the US. It aims at addressing organizational capacities, structures, cultures, and processes that are relevant to further developing hospitals into health literacy-friendly settings [29]. In contrast to the US 10 Attributes, this concept follows the theory-based comprehensive health-promotion settings approach [47], ISQUA quality methodology, and a wider definition of health literacy [23,29]. The bOJA/BÖJI working group used the V-HLO concept as a blueprint and adapted it for settings of open youth work. These concepts were chosen because (1) they were already available in the German language, (2) they provide many organizational health-literacy dimensions that are relevant to our own project agenda, and (3) they were developed in collaboration with stakeholders in practice. This was perceived as fundamentally important to increase user involvement and ensure that the concept meets the needs of schools regarding health literacy. The bOJA/BÖJI-concept, especially, was regarded as highly relevant and suitable to be adapted within the HeLit-Schools project. This is because the fields of extracurricular youth work and educational work in schools have the same target group, with both settings playing a significant role in the lives of young people. In addition, both settings show parallels regarding educational content and responsibilities, even though the conditions of the settings partially differ. Furthermore, the development of the HeLit-Schools concept was guided by the methodological approach used in that project.

### 2.2. Adaptation Procedure

The planned procedure originally involved on-site workshops in which central stakeholders of the health sector, health-literacy research, and those working in the context of school and education could take part in the adaptation process and share their expertise. This was changed as the COVID-19 pandemic restricted possibilities for meeting. In order to benefit from the expertise of relevant stakeholders such as teachers, principals, practitioners, and researchers, they were asked to comment, review, and validate the developed conceptualization at different stages of the adaptation process.

The project staff developed a first draft version of the HeLit-Schools concept. The two reference concepts mentioned above were analyzed and discussed in-depth. All dimensions, as well as the concepts as a whole, were examined to identify how the corresponding aspects must be adapted in order to fit the framework and reality of the school setting. Changes were made to make the concept more applicable to schools: Some aspects were deleted and some added, according to the specifics of the school setting, as described in the following. Additionally, the wording was critically reviewed and adjusted to fit to schools.

The first draft of the concept describing standards of the health-literate school, including indicators specifying contents and objectives of every standard, was sent to project partners and advisory board members from ministries, academia, and practice to be reviewed and commented on. Their feedback was reviewed and assessed independently by two members of the project staff following a discussion of necessary and well-founded modifications. After reaching consensus, modifications were made. This procedure was repeated every time new comments were received. Comments from the partners and advisory board members mainly targeted the conceptual structure of the standards and indicators, which led to reduction and merging of standards, revision of indicators, and removal of sub-indicators.

A modified version of the concept was then presented and discussed at a meeting of the Interdisciplinary Centre for Health Literacy Research at Bielefeld University with health literacy and school experts from academia. The project team collected comments, recommendations and critical remarks, subsequently reviewing them in order to update the concept draft. The updated version was again sent for review by principals of schools in Bielefeld. This feedback loop revealed modification needs regarding educational and school-related content. Modifications were requested for aspects such as organizational processes, tasks of school supervisors and school boards, and interaction with the immediate school environment and parents, which had been previously unconsidered or described imprecisely. Recommendations were made regarding the allocation of indicators to standards. Some aspects, for instance, are located within the responsibilities of school principals (definition of the school’s mission statement and philosophy, support of health promoting measures, communication with authorities), whereas other aspects structurally belong to the standard that addresses school development (use of resources, school development plans, extracurricular activities, in-service teacher training). Furthermore, suggestions were made, e.g., regarding offers and activities for parents (such as parents’ evenings), teacher trainings, the depiction of functions in the school, as well as the linking of school content and topics of health promotion and prevention already embedded in everyday school life. Critical remarks pointed at unrealistic requirements within the concept with regard to tasks for strengthening health literacy. In particular, the underlying, but not intentional, expectation that schools must address all these aspects simultaneously and on their own needed to be clarified, as the concept is meant to serve as a guideline for school development.

During the adaptation process, our Austrian project partners from “Styria vitalis” developed and released the guide “Schools for Health Literacy” [36]. As the work of Styria vitalis is based on the same concepts of organizational health literacy that we used in the HeLit-Schools projects (bOJA/BÖJI-concept and V-HLO), we decided to analyze their concept in order to refine our own. This guideline contributed to the further refinement of our concept and, in particular, led to a simplification of the HeLit-Schools concept and the reduction of indicators.

## 3. Results

The HeLit-Schools concept resulted from the adaptation work. This concept offers a comprehensive approach for the organizational development of schools into health-literate schools. Within the concept, a health-literate school is defined as follows: 

A health-literate school optimizes processes, structures and frameworks in such a way that health literacy can be developed, practiced and enhanced within and through its setting. A health-literate school enables everyone involved in the school—students, school principals, teachers and non-teaching staff, parents and caregivers as well as persons in the extended school environment—to deal with and manage health information and to improve and reinforce health-literate behavior [37].

In order to enhance health literacy in the whole school, the concept builds on both behavior and social change and addresses different aspects of the school organization crucial to processes of organizational change. From a systems and organizational perspective, schools are complex, socio-ecological systems [40] that can have a significant influence on everyone at or related to the school. Therefore, it is essential to consider all relevant areas of organizational development in order to adequately meet the complexity of the school organization and its health-promoting potential [44,48]. The main benefit of this approach is that improvements of the socio-ecological system, i.e., focusing on the school in this case, will facilitate improvement of individual behavior. In this context, this means that improving organizational health literacy will support the enhancement of students’ personal health literacy.

Developing a school into a health-literate organization can best be embedded into school development processes involving systematic planning and implementation of structural and cultural changes. In doing so, different levels need to be considered in a complementary manner as they are reciprocal. According to theoretical models describing school development processes, these levels address (i) organizational development, (ii) instructional development, and (iii) the school staff development [40]. In addition, schools are only one component influencing and determining the living environment next to other systems (e.g., the family). Accordingly, the wider school environment represents a fourth relevant level (iv) that also needs to be considered in a holistic approach to school development regarding health literacy and health [44,49].

### HeLit-Schools Concept

The HeLit-Schools concept comprises eight dimensions of development areas which are called “standards” and can be understood as guidelines for a health-literate school. These standards address the four developmental areas referred to above and fulfill the requirements of a holistic health promoting setting approach to school development. Each standard comprises six measurable indicators that further describe the objectives and contents of the respective standard. The standards, including a brief summary of the indicators, are presented in Table 1.

The standards and indicators of the HeLit-Schools concept are the basis for a questionnaire that assesses the organizational health literacy of schools. Within the questionnaire, the six measurable indicators are presented for every standard, accompanied by a four-point scale to insert the respective rating and an open box for comments and notes. Thus, the questionnaire functions as a self-assessment tool. It guides a school’s evaluation of its health-literacy-related capacities: Which structures, framework conditions, processes, cultures, etc., already foster health literacy in everyday school life? Where are the gaps and starting points for planning and realizing changes to further develop the school into a health-literate school? This questionnaire will be published and made available for schools to use in the near future.

## 4. Discussion

The HeLit-Schools concept describes a holistic approach to organizational development regarding the strengthening of health literacy in schools. It includes measures on the individual level in terms of behavior change, and measures on the organizational level of the school in terms of social change. When we started our project and the development of this concept in Fall 2019, it was, to our knowledge, the first comprehensive concept of organizational health literacy for schools. Parallel to our project, our colleagues from “Styria vitalis” in Austria developed a similar concept, also based on the V-HLO and bOJA/BÖJI concepts to be used by schools in Austria [36]. 

Within the standards, all relevant levels of school development mentioned above are represented (school staff, teaching, organization, wider school environment). These also reflect common fields of action of school health-promotion approaches [4]: Standards 3, 4 and 5 focus on strengthening the personal health literacy of students and the school team. Thus, they are located within the individual field of action. Strengthening personal health literacy among students in the school setting has been emphasized internationally for some time [1,4,9,19,50]. In addition, the school team is an important addressee for health literacy promotion: school principals, due to their key function in school development and the facilitation of health promotion, are crucial for fostering health literacy at schools [51,52]; and teachers, as they are central actors regarding learning and interaction [1].

Standards 1, 2 and 6 can be placed within the field of action of the school level. Previous work regarding school health promotion, but also organizational health literacy, indicate that environmentally oriented approaches are promising. This is because changes at the system level are considered more sustainable and more effective than solely addressing the individual level [4,19].

Finally, Standards 7 and 8 are directed at the wider school environment. Schools do not act as isolated organizations but are rather integrated into wider community systems. Therefore, the cooperation of local organizations, and services, etc., is described as a significant factor within the community field of action to promote health literacy in schools [1,4].

To further develop a school into a health-literate organization can be seen as rewarding, but also demanding, as the approach includes adjustments within the entire school organization. While the presented benefits offer sustainability and effectiveness regarding the early promotion of health literacy, the practical implementation can be seen as challenging. Systematic efforts on different levels as well as time, personnel, and financial resources are required.

To support schools in becoming more health-literate, the HeLit-Schools project also aims to provide different tools based on the HeLit-Schools concept: an implementation guideline, a collection of existing materials and programs to strengthen health literacy in the school setting, and a self-assessment tool to measure schools’ organizational health literacy. These tools are intended to guide their organizational development: from baseline inventory to developing and implementing changes and measures, and finally, reviewing the development process. As of now, the development of these tools has been completed and they will be published shortly.

Currently, there is not yet an empirical basis with regard to practicability and implementation processes of the concept in schools. Thus, there is no detailed information about facilitators and barriers. However, the HeLit-Schools project goals also include the measurement of the organizational health literacy of schools. School principals will be invited to assess their school using the developed questionnaire. Analysis of the generated data promises empirical validation of the concept and insights into potentials and necessities across schools regarding the organization-based, systemic strengthening of health literacy in the school setting. After identifying supporting factors and barriers, these will be transferred into practical recommendations.

## 5. Conclusions

As the importance of health literacy grows, settings-based interventions and approaches are much needed in order to foster personal health literacy more effectively. Schools can play a crucial role in enhancing the health literacy of students and virtually every individual at the school. The HeLit-School concept is based on the organizational health-literacy approach adopted to the school setting. The concept guides the organizational development of schools in order for them to become health-literate organizations. The concept includes a combination of skill-promoting measures in terms of behavior change and measures for altering the structures and organizational conditions of the school and its entire environment in terms of social change. Thus, this holistic concept, originally developed for schools in Germany, seems to be a promising approach for effectively and sustainably strengthening health literacy in and through schools, including those beyond Germany in the wider international context. 

## Figures and Tables

**Table 1:** Eight standards of a health-literate school (HeLit-Schools concept).

Standard	Description of Objectives and Contents
1. Include health literacy into the school’s mission statement	Health literacy becomes part of the school’s mission statement. Promoting and enhancing health literacy is included as an important goal of school health promotion and prevention. Subsequently, school principals, teachers and school staff acknowledge health literacy to be important and facilitate measures to improve health literacy in the whole school. In order to promote the implementation of standard 1, school principals are particularly significant as they are crucial for school changes. Additionally, school authorities and district board administrations are a major factor for supporting and facilitating health literacy activities in the school, as they can enable the allocation of necessary monetary funding and resources.
2. Health literacy as part of school development	Health literacy is included as an agenda item at the level of school development. Measures fostering health literacy in and outside the classroom should be implemented and further developed. A delegation of school staff should be appointed responsible for leading and driving activities related to health literacy. In addition, personnel, time and financial resources need to be made available.
3. Promote and enhance health literacy in daily school life	Effective promotion and enhancement of health literacy requires making changes to daily school life and associated processes. This includes (re-)organizing and modifying existing teaching content, methods, materials, and instructions, and providing accessible, age-appropriate, real-world and diversity-sensitive health information. In addition, health-literate action and health behavior by the school team must be considered as they function as role models for students. The goal is to help students to develop, enhance, and practice health literacy.
4. Health literacy of students	Standard 4 is dedicated to promoting and enhancing personal health literacy of students at the classroom and school levels, e.g., by providing learning opportunities. Health literacy should be embedded into existing activities of school health promotion and prevention and as a cross-cutting topic to other subjects, e.g., in the context of digital education/digital literacy, media education/media literacy, or physical activity. Teaching materials and content should be available and used to equip students with health knowledge and skills to understand, think critically and apply health information from different sources and in different contexts. As students’ participation is key, their interests and needs should be considered regarding the selection and design of health content.
5. Health-literate school staff	Standard 5 focuses on strengthening personal health literacy of school principals, teachers and school staff. Education and training opportunities should be made available and taken advantage of. In addition, the health of the school team must be addressed and enhanced by health promoting measures and improvements in the school environment.
6. Health-literate communication at school	A health-literate school emphasizes the importance of simple, clear and understandable communication. Within the school and beyond, e.g., in conversations with parents or guardians, communication regarding health issues should be simple and understandable for everyone. Additionally, sensitivity for communication about health topics (physical and mental health) should be created and corresponding skills should be promoted in the classroom and in daily school life (e.g., critical thinking, communicating in a way that facilitates understanding). Appropriate communication methods should be applied and teacher trainings regarding the topics of communication and health should be provided.
7. Enhance health literacy in the school environment	Standard 7 is dedicated to enhancing health literacy in the whole school environment, e.g., within the framework of school health promotion and prevention. Within the school, health literacy should be used to achieve school health promotion goals. Looking externally, the school should cooperate with various school and non-school (health-related) actors and support systems, function as a (first) point of contact, facilitator, and mediator for their students’ health-related concerns and involve parents and caregivers as relevant partners regarding issues of school health.
8. Networking and cooperation	Standard 8 is dedicated to networking, cooperation and exchange regarding health literacy in the proximal and distal school environment and related school community. Networking and cooperation with health-related actors (e.g., healthcare providers, doctors, nurses, mental health professionals) as well as parents and caregivers are essential for sharing knowledge and experience and making health literacy visible both inside and outside the school.

## Data Availability

Not applicable.
